# Supplementary material for: Evaluation of Pretreatment Albumin–Bilirubin Grade as a Better Prognostic Factor Compared to Child–Pugh Classification in Patients with Hepatocellular Carcinoma Receiving Transarterial Chemoembolization Combined with Radiotherapy
Source: J Pers Med. 2023 Feb 17;13(2):354. doi: 10.3390/jpm13020354 (PMC9963241; doi:10.3390/jpm13020354)
Supplement: Supplementary file 1 [file jpm-13-00354-s001.zip › jpm-2189206-supplementary.pdf]

Supplementary Table S1-1. The detailed relationship between pretreatment ALBI* grade and Child-Pugh score

ALBI grade	Child-Pugh score	No. of patients (%)	*p* value
1 (n=33)	5	30 (90.9)	<0.001
	6	3 (9.1)	
2-3 (n=40)	5	10 (25.0)	
	6	21 (52.5)	
	7	8 (20.0)	
	8	1 (2.5)	

Supplementary Table S1-2. The detailed relationship between pretreatment Child-Pugh grade and ALBI score

Child-Pugh grade	ALBI score (median, range)	*p* value
A (n=64)	-2.61 (-3.29--1.67)	<0.001
B (n=9)	-2.11 (-2.30--0.89)	

* Albumin-bilirubin

Supplementary Table S2. Univariate and multivariate Cox proportional hazards associations between clinical factors and survival outcomes

	Progression-free survival				Overall survival			
	Univariate analysis		Multivariate analysis		Univariate analysis		Multivariate analysis	
Variable	HR (95% CI)	*P* value	HR (95% CI)	*P* value	HR (95% CI)	*P* value	HR (95% CI)	*P* value
Age at TACE+RT ≤60 vs > 60	0.571 (0.338-0.964)	0.036	0.709 (0.405-1.240)	0.228	1.029 (0.549-1.927)	0.930		
Gender	1.122 (0.346-3.632)	0.848			2.077 (0.633-6.818)	0.228		
ECOG 0-1vs2-3	1.201 (0.680-2.122)	0.527			1.399 (0.696-2.818)	0.346		
mUICC stage 1-3 vs 4A	1.640 (0.997-2.698)	0.051	1.374 (0.812-2.325)	0.237	2.703 (1.374-5.319)	0.004	2.449 (1.236-4.854)	0.010
Pre-TACE AFP (ng/mL) ≤80vs>80	1.518 (0.921-2.503)	0.102			1.108 (0.597-2.059)	0.745		
Pre-TACE PIVKA-II (mAU/mL) ≤496.5vs>496.5	1.305 (0.748-2.278)	0.349			1.150 (0.558-2.370)	0.705		
Pre-TACE Child-Pugh grade	1.503 (0.727-3.107)	0.272			1.222 (0.540-2.768)	0.631		
Pre-TACE ALBI score	2.319 (1.343-4.007)	0.003	2.073 (1.188-3.617)	0.010	2.814 (1.463-5.412)	0.002	2.463 (1.278-4.749)	0.007

Abbreviations: HR, Hazard ratio; CI, Confidence interval; TACE, Transarterial chemoembolization; RT, Radiotherapy; ECOG, Eastern Cooperative Oncology Group; mUICC, modified Union for International Cancer Control; AFP, Alpha-Fetoprotein; PIVKA-II, Prothrombin induced by vitamin K absence-II; ALBI, Albumin-bilirubin.

Supplementary Table S3. Pattern of change in ALBI grade after the treatment

Before TACE+RT	1-2 months after TACE+RT	No. of patients (%)
ALBI grade 1 (n=33)	ALBI grade 1	21 (63.6)
	ALBI grade 2	12 (36.4)
ALBI grade 2 (n=39)	ALBI grade 1	6 (15.4)
	ALBI grade 2	28 (71.8)
	ALBI grade 3	5 (12.8)
ALBI grade 3 (n=1)	ALBI grade 2	1 (100.0)

Abbreviations: TACE, Transarterial chemoembolization; RT, Radiotherapy; ALBI, Albumin-bilirubin.
